# Recovery and maintenance of NESTIN expression in umbilical cord-MSC using a novel culture medium

**DOI:** 10.1186/s13568-020-01067-7

**Published:** 2020-07-28

**Authors:** Yuncheng Liu, Feidi Xiao, Xiang Hu, Zan Tang, Zeqin Fu, Xiao Liang, Guifang Zeng, Weijie Zeng, Yan Liao, Yuan Ren, Zhiyu Liu, Hao Peng, Qiuhong Mei, Muyun Liu

**Affiliations:** 1 National-Local Associated Engineering Laboratory for Personalized Cell Therapy, Nanshan, Shenzhen, Guangdong People’s Republic of China; 2 Shenzhen Beike Biotechnology Co., Ltd., Nanshan, Shenzhen, Guangdong People’s Republic of China; 3 Shenzhen Kenuo Medical Lab, Nanshan, Shenzhen, Guangdong People’s Republic of China

**Keywords:** NESTIN, UC-MSC, Culture medium, UltraGRO

## Abstract

Mesenchymal stem cells (MSC) are a popular candidate in cellular therapy for many diseases. MSCs are well known by their feature of self-renewal and their differentiation potential. NESTIN is a cytoskeletal protein expressed in MSC that functions directly in cell proliferation and differentiation. Here, we demonstrated that adding UltraGRO, a medium supplement, could maintain and partially recover the expression of *NESTIN* in human umbilical cord derived MSCs (UC-MSCs). Furthermore, the UC-MSCs cultured with UltraGRO showed a better immunomodulation ability in a colitis mouse model compared with those cultured in other types of media. This indicates that the use of novel culture medium benefits the maintenance of *NESTIN* expression and NESTIN may be one of the vital factors that regulates the performance of MSCs.

## Key points

1. NESTIN affects the proliferation and differentiation of mesenchymal stem cells, however, its expression decreases over time with frequent, iterated passaging.
2. Using a novel culture medium by adding UltraGRO can sufficiently maintain and recover the expression of *NESTIN* in umbilical cord MSCs for up to 10 passages.
3. The UC-MSCs cultured using this novel medium have better immunomodulatory ability in colitis mice models.

## Introduction

Since Friedenstein and co-workers first identified the differentiation potential of bone marrow cells in 1987, numerous studies on stem cells have been conducted in various fields. These studies have demonstrated that “bone marrow fibroblasts” and their descendants originated from non-hematopoietic stem cells and could differentiate into mesenchymal cells of other tissues (Friedenstein et al. 1987). So-called “mesenchymal stem cells” (MSCs) are widely studied mainly due to their feature of self-renewal and their differentiation potential (Uder et al. 2018). The International Society of Cell Therapy (ISCT) specifically defines human MSC by three criteria. The first is that MSCs can adhere to plastic surfaces, which is utilized to easily isolate cells from tissues under standardized culture conditions. Second, MSCs from different sources must have a similar combination of surface markers, that is, up to 95% of the MSC population expresses CD105, CD73, and CD90. However, less than 2% of MSC may express CD45, CD34, CD14 or CD11b, CD79 or CD19 and HLA-DR (Dominici et al. 2006). Lastly, MSCs must feature a robust differentiation potential, and must be able to differentiate into at least three different lineages in vitro, such as osteoblasts, adipocytes, and chondrocytes (Uder et al. 2018). These criteria have become a gold standard in the stem cell therapy industry, and are used to confirm MSC during all manufacturing processes, from cell isolation to stimulated culture and finally to large-scale expansion.

Despite MSCs first being identified from bone marrow, they have also been isolated from many other tissues and organs, such as muscle, thymus, pancreas, adipose, dental pulp, umbilical cords, and placenta (Ferrari et al. 1998; Seo et al. 2004; Zuk et al. 2001). MSCs are distributed in many different organs likely because MSCs are generated in and can migrate via large and small blood vessels. This may also be relevant to their residential preference in perivascular niches throughout post-natal organisms (da Silva Meirelles et al. 2006). Accordingly, MSCs are manufactured by adipose tissue, umbilical cords, and placenta, which are commonly treated as clinical wastes, under current Good Manufacturing Practice (cGMP) in the stem cell therapy industry. However, flow cytometry analyses revealed that MSCs between different sources and species have similar but not identical surface markers and functions (Donnenberg and Ulrich 2013). Thus, investigations of the expression of surface markers and morphology were performed to monitor the association of effects on variability in terms of isolation yield, proliferation rates, and expansion ability (Kern et al. 2006; Oedayrajsingh-Varma et al. 2006; Seo et al. 2005).

NESTIN, a neuroepithelial stem cell protein, is also a cytoskeletal protein expressed in stem cells (Lendahl et al. 1990; Mignone et al. 2004). It is believed that NESTIN functions in the stem cell processes of self-renewal, proliferation, differentiation, and migration (Bernal and Arranz 2018; Kulkarni et al. 2017; Mignone et al. 2004). According to the gene sequence and protein structure homology, NESTIN is classified as a type VI intermediate filament that contributes to cytoskeleton constitution (Bernal and Arranz 2018). The constitutive expression of *NESTIN* by undifferentiated MSC is regarded as a marker of the “multi-differentiated” state in which cells can retain their neuronal differentiation property (Tondreau et al. 2004). Moreover, *NESTIN* expression is developmentally regulated because it is inversely correlated with cell differentiation (Wiese et al. 2004). NESTIN is down-regulated by the transition from proliferating neural stem cells to post-mitotic neurons with specific stimulus (Wiese et al. 2004; Zimmerman et al. 1994). Many recent studies have indicated that NESTIN may be an important reporter of cell state related to proliferation and differentiation potential of MSCs (Bernal and Arranz 2018; Lu et al. 2019; Wong et al. 2014).

However, NESTIN is not currently a common selective marker for MSCs in either ISCT criteria or the stem cell therapy industry. MSCs are prominent due to their self-renewal potential, which allows the propagation of MSCs in vitro under specific isolation and cultivation procedures (Uder et al. 2018). Since MSC application for clinical use has strict requirements for sufficient cell number and consistent cell quality along the whole cultivation process, the in vitro expansion of MSCs has been extensively investigated. These studies revealed that the isolation, culture, expansion procedures and cell differentiation during the manufacturing process are possible factors resulting in the inconsistency (Lodie et al. 2002; Uder et al. 2018). The conventional MSC culture protocol as described in numerous studies uses Dulbecco’s modified Eagle’s medium (DMEM) with 10% fetal bovine serum (FBS). Since frequent iterated passaging for MSC would lead to cell aging and loss of stemness, human MSC culture is confined prior to the 6th passage in order to maintain the unrestricted differentiation capacities (Bonab et al. 2006; Halfon et al. 2011).

The ex vivo expanded MSCs have a potent immunomodulatory function in the treatment of immune disorders. Many inflammation-related cytokines, such as interleukin-6 (IL-6), interleukin-1β (IL-1β), indoleamine 2,3-dioxygenase (IDO) and prostaglandin E2 (PEG2), can be modulated in the presence of MSCs (Chinnadurai et al. 2014; Lara et al. 2017; Wang et al. 2017). The clinical potential of MSC transfusion has been explored in several animal models of immune disorders including autoimmune encephalitis, rheumatoid arthritis, transplant rejection and Crohn’s disease (Ren et al. 2012; Uccelli et al. 2008). In this study, we monitored the *NESTIN* expression in umbilical cord MSCs (UC-MSCs) in vitro for up to ten passages using three different types of culture media. Moreover, we verified the immunomodulatory ability of UC-MSCs in colitis mice models.

## Materials and methods

### Isolation and culture of human UC-MSCs

Institutional review board approval from the Shenzhen Integrated Cell Bank was obtained for all procedures. Fresh umbilical cords (UC) were collected for scientific study from three healthy donors who were informed and consented. Mesenchymal tissue was scraped from Wharton’s jelly after blood vessels were removed (Devito et al. 2019). After cutting it into pieces, the tissue was centrifuged at 600*g* for 10 min at room temperature. Tissue was then washed with 0.9% saline and cultured at 37 °C with 5% CO_2_ in serum-free Dulbecco’s modified Eagle’s medium (DMEM). The primary UC-MSCs (P0) were obtained after 10 days of culture. A sufficient number of MSCs from the same source was equally divided into three groups in order to ensure the same starting conditions. During the 10-passage culture, UC-MSCs were plated at the density of 5 × 10^3^ cells/cm^2^ into flasks for each group and cultured at 37 °C and 5% CO_2_. Three culture medium supplements were applied for comparison including fetal bovine serum (FBS), fetal bovine serum substitute (FS) (TBD, China), and UltraGRO-Advanced (Helios Bioscience, UK); the base medium for all cultures was DMEM/F-12 (Gibco, US). Base culture ingredients were DMEM and 10% FBS, which is the conventional culture medium. The second type of media was DMEM with 2% TBD FS and 1% GlutaMax, and the third was DMEM with 5% UltraGRO-advanced and 1% GlutaMax.

### Induction assay for UC-MSC differentiation

Based on a published procedure, the UC-MSCs were induced to differentiate into osteogenic, adipogenic and chondrogenic stem cells in vitro (Lei et al. 2013).

### UC-MSC assay proliferation

To investigate the effects of different culture systems, 10th passage UC-MSCs were used to perform the following experiments. The 10th passage UC-MSCs of the three groups were passaged and the cell counts were recorded at the 6th, 12th, 24th, and 48th hours of culture. The expansion ratio was calculated by dividing the total cell count by the initial plated cell number. Moreover, 10th passage UC-MSCs of the three groups were also treated with 200 ng/ml IFN-γ for 4 h. IDO and PGE2 were detected in the culture supernatant using an ELISA from BioLegend (San Diego, CA).

### Flow cytometry analyses

Flow cytometry analyses were performed using a FACSaria™ III cytometer (BD Bioscience, San Jose, CA) and the data were analyzed with the FlowJo7.5 (Treestar, Ashland, OR) software packages. In order to identify UC-MSCs, Antihuman CD29-PE (MAR4), CD73-PE-CyTM7 (AD2), CD90-FITC (5E10), CD105-APC (266), CD34-PE (563), CD45-FITC (HI30), CD14-FITC (M5E2), CD79α-APC (HM47) and HLA-DR-PreCP (G46-6) antibodies, along with the corresponding isotype control antibodies were purchased from BD Pharmingen. To monitor the *NESTIN* expression, antihuman NESTIN-APC (IC1259A) antibody along with the corresponding isotype control antibody was also purchased from R&D Pharmingen. As NESTIN is a protein of the cytoskeleton, cell fixation and permeabilization were performed before cell staining for flow cytometry. Sufficient MSCs from each group at each passage were harvested for flow cytometry analyses.

### Quantitative PCR assay

UC-MSC samples were collected at each time point and stored at − 80 °C. RNA extraction was then performed using the RNeasy Mini Kit from QIAGEN. The forward and reverse primers for qPCR were designed for *NESTIN* detection (F: CTCCAAGAATGGAGGCTGTAGGAA, R: CCTATGAGATGGAGCAGGCAAGA). QuantiFast®SYBR® Green PCR Kit from QIAGEN was used to perform qPCR.

### Experimental colitis induced by TNBS

All of the animal procedures were reviewed and approved by the Beike Animal Care and Use Committee. For inducing colitis in 9-week-old male BALB/c mice, we referred to a previously published protocol (Wirtz et al. 2007). On day 1, mice were smeared with 200 µl of a pre-sensitization solution of trinitrobenzene sulfonic acid (TNBS; Sigma) on their backs. On day 8, mice were divided into ten groups (8 mice/group) and fasted (but allowed to drink ad libitum) for 24 h. On day 9, mice were weighed and treated intrarectally with 150 µl 3% TNBS in saline (eight groups) and no solution (blank control, two groups). At 10 h post TNBS injection, animals were transplanted i.p. with 500 µl saline, or 4 × 10^6^ MSCs from the three groups that were suspended in 500 µl saline. Colons were collected from the caecum to the anus on day-1, day 0, day 2, day 4, day 6 and day 10 after TNBS injection.

### Cytokine assays

The culture supernatant of 10th passage UC-MSCs was used to detect the cytokine production of IDO (DY6030-05) and PGE2 (KGE004B) with an ELISA from R&D system Inc. Mouse serum was used to detect the cytokines. IL-6 (431304) and IL-1β (437003) were analyzed using an ELISA from BioLegend (San Diego, CA) and SOD (MAB3419-SP) using an ELISA from R&D system Inc.

### Statistical analysis

Statistical comparisons were performed using the two-tailed Student’s *t*-test (between two groups) or a one-way analysis of variance (ANOVA). P < 0.05 was considered to represent a significant difference. Analysis and graphing were performed using WPS office software.

## Results

**Fig. 1:**
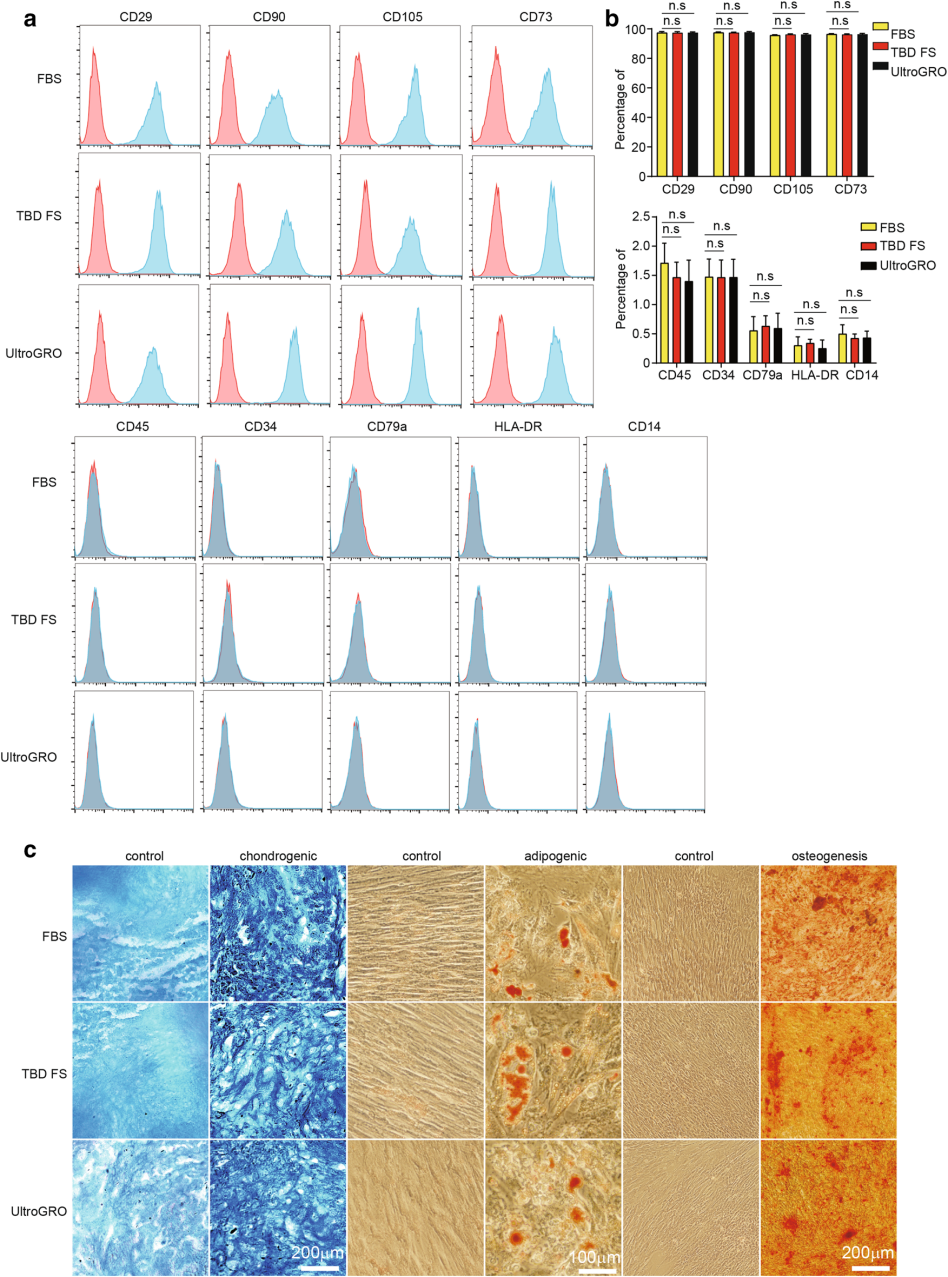
The characterization of UC-MSCs in three different culture conditions. **a** The UC-MSCs at the 10th passage in three culture conditions were positive for CD29, CD90, CD105, and CD73, and were negative for CD45, CD34, CD79a, HLA-DR, and CD14. **b** The flow cytometry statistics of stem cell markers in three culture conditions. **c** The UC-MSCs at the 10th passage in three culture conditions were stained with Alcian blue, oil red O, and Alizarin Red S, respectively. For all experiments, n = 3 healthy donors/group, and data shown are mean ± SD.

The three different culture conditions used to obtain mesenchymal stem cells (MSCs) for the comparisons were: DMEM-F12 with 10% fetal bovine serum (FBS), DMEM-F12 with 10% fetal bovine serum substitute (FS), and DMEM-F12 with 5%UltraGRO-Advanced. UC-MSCs were harvested at various passages throughout the culture period, and the immunophenotype of the MSCs was investigated via quantitative flow cytometry (Uder et al. 2018). Flow cytometric analyses revealed that the UC-MSCs at the 10th passage in all three culture conditions expressed representative surface markers, including CD29, CD73, CD90, and CD105, but not CD34, CD45, CD14, CD79, or HLA-DR (Fig. 1a). Cells from the 2nd to 9th passage also had the same surface marker repertoire (data not shown).To compare the differentiation potentials, the UC-MSCs in different culture conditions were induced to differentiate into adipogenic, osteogenic, or chondrogenic lineages. UC-MSCs in all three culture conditions at the 10th passage were successfully induced to differentiate into adipogenic, chondrogenic, and osteogenic cells (Fig. 1b), and the UC-MSCs from the 1st to 9th passage had the same differentiation ability. The results suggest that all three culture conditions could sustain the surface marker repertoire and differentiation potential of the UC-MSCs from the 1st to 10th passage.

**Fig. 2:**
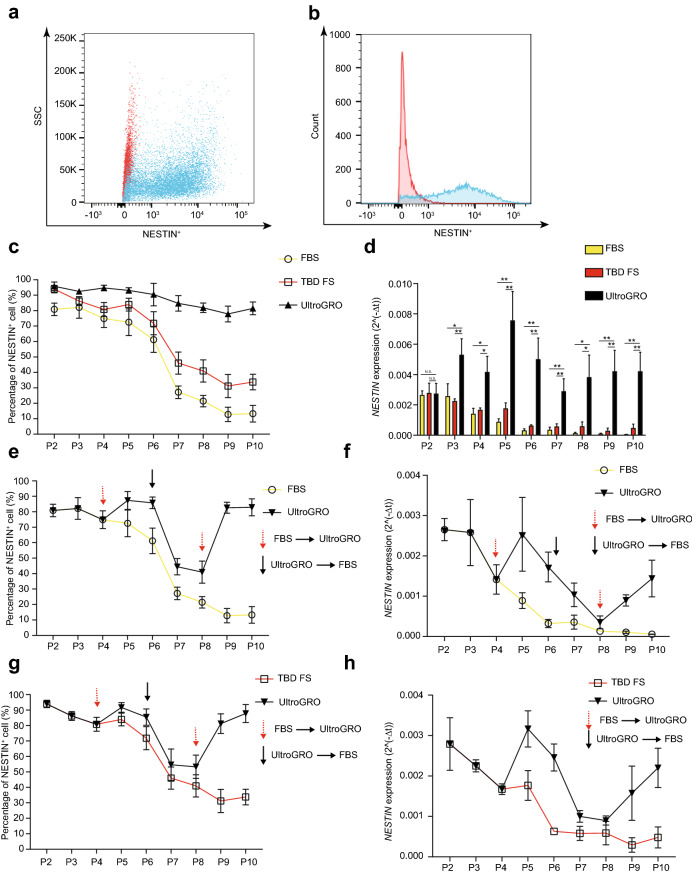
The expression of *NESTIN* in three different culture conditions. **a**, **b** The flow cytometry analysis of NESTIN in UC-MSCs. **c** The percentage of NESTIN^+^ cells in three culture conditions. **d** The expression level of *NESTIN* in three culture conditions. **e**–**h** The percentage of NESTIN^+^ cells and expression level of *NESTIN* after changing the medium. **p < 0.01, * p < 0.05, Student’s *t*-test. For all experiments, n = 3 healthy donors/group, and data shown are mean ± SD.

We next examined the percentage of NESTIN^+^ UC-MSCs by flow cytometry analysis (Fig. 2a, b) and the *NESTIN* expression level in UC-MSCs by quantitative reverse transcription PCR (RT-qPCR) in three different culture conditions in each passage. The ratio of NESTIN^+^ UC-MSCs could be stably maintained before the 6th passage in all three culture conditions. However, the percentage of NESTIN^+^ UC-MSCs significantly decreased starting at the 7th passage in the other two culture conditions compared with UltraGRO-medium (Fig. 2c). Consistently, NESTIN in UC-MSCs in three different culture conditions had a similar trend with the exception of expression level (Fig. 2d). *NESTIN* in UltraGRO-medium was expressed at a relatively higher level than the others. Despite the fact that UltraGRO-medium could sustain the percentage of NESTIN^+^ UC-MSCs, we further investigated whether the shift of UltraGRO-medium could recover the expression of *NESTIN* in UC-MSCs in the other two culture conditions. The UC-MSCs were initially cultured in FBS-medium or TBD-FS-medium, and then changed to UltraGRO-medium at the 4th passage. We found that the percentage of NESTIN^+^ UC-MSCs increased after changing medium compared with the UC-MSCs cultured using consistent culture conditions. If we changed the culture medium back to FBS-medium or TBD-FS-medium at the 6th passage, the percentage of NESTIN^+^ UC-MSCs declined significantly and increased significantly when the medium was changed back to UltraGRO-medium at the 8th passage (Fig. 2e, g). The expression level of *NESTIN* is also consistent with this trend (Fig. 2f, h). These results indicated that UltraGRO-medium could not only maintain but also recover the expression of *NESTIN* in UC-MSCs.

**Fig. 3:**
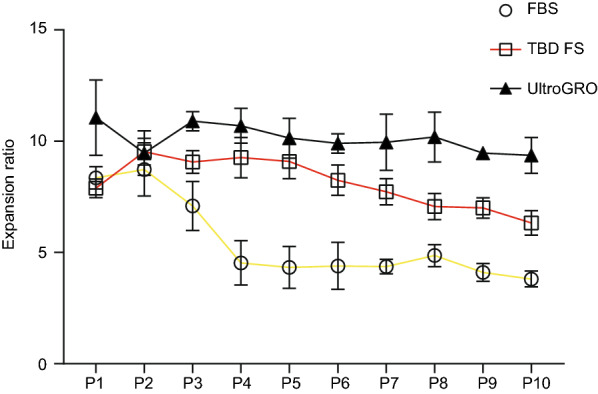
The proliferation capacity of MSCs in the three groups. The proliferation ability of UC-MSCs in FBS, TBS FS, and UltraGRO group was measured by cell count. For all experiments, n = 3 healthy donors/group, and data shown are mean ± SD.

We then evaluated the proliferation capacity of UC-MSCs under different culture conditions. The expansion ratio of the UC-MSCs cultured in three different culture conditions was calculated between the starting cell density (5 × 10^3^/cm^2^ in a 175 cm^2^ flask) and the cell density every 2 days. UC-MSCs cultured in UltraGRO-medium sustained their proliferation capacity with an expansion ratio above ten in each passage. However, the expansion ratio of UC-MSCs cultured in FBS-medium or TBD-FS-medium gradually decreased (Fig. 3). This result suggested that UltraGRO-medium could sustain a greater proliferation capacity of UC-MSCs than medium with the other two culture supplements.

**Fig. 4:**
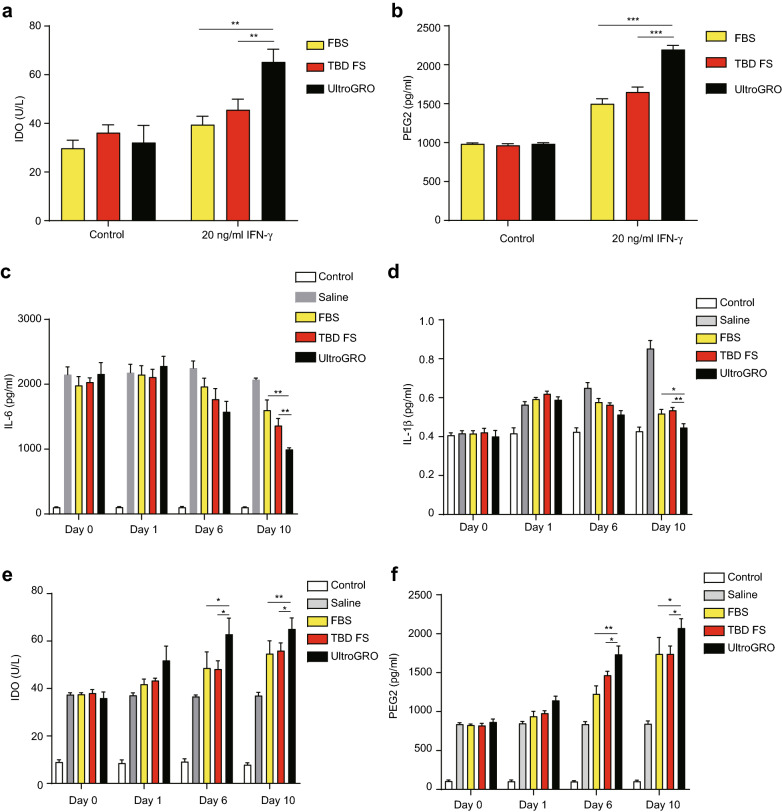
The measurement of UC-MSCs secreted cytokines in vitro and in vivo. **a**, **b** The anti-inflammatory cytokines IDO and PEG2 secreted by UC-MSCs were measured by ELISA after being treated with 200 ng/ml IFN-γ. **c**, **d** The IL-6 and IL-1β were measured by ELISA in a TNBS-induced colitis mouse model. **e**, **f** The IDO and PEG2 were measured by ELISA in aTNBS-induced colitis mouse model. **p < 0.01, * p < 0.05, Student’s *t*-test. For all experiments, n = 3 healthy donors/group, and data shown are mean ± SD.

To evaluate the immunomodulation ability of UC-MSCs that highly express *NESTIN*, we treated the 10th passage UC-MSC in all three types of media with IFN-γ, and measured the IDO and PEG2 secretion using an ELISA. IDO and PEG2 were significantly increased after IFN-γ treatment. Furthermore, the IDO and PEG2 levels were significantly higher in the UC-MSCs cultured with UltraGRO than with the other two supplements (Fig. 4a, b). In order to analyze the immunomodulation ability in vivo, we intraperitoneally injected UC-MSCs into a trinitrobenzene sulfonic acid (TNBS)-induced colitis mouse model, and measured the proinflammatory cytokines and anti-inflammatory cytokines using an ELISA. The pro-inflammatory cytokines (IL-6 and IL-1β) were down-regulated after UC-MSC injection, and UC-MSCs cultured in UltraGRO-medium showed a better inhibition effect than in the other two media (Fig. 4c, d). The anti-inflammatory cytokines (IDO and PEG2) were up-regulated simultaneously (Fig. 4e, f). This indicated that UC-MSCs may contribute to the immunomodulation against colitis and the UltraGRO group presents the best performance in immunomodulation among the three different culture conditions.

## Discussion

The differentiation and proliferation capacity of UC-MSC weakens as frequent, iterated passaging increases especially after 40- to 50-fold population doubling (Halfon et al. 2011). Furthermore, the loss of stem cell features after the 6th passage was coincident with the decline of the mean telomere length from 9.19 kb to 8.7 kb at the 9th passage (Bonab et al. 2006). Previous studies have demonstrated that the expression of *NESTIN* is essential in cellular differentiation and regulation, and that it produces progenitor cells (Wiese et al. 2004; Wong et al. 2014). However, there are no studies tracking the connection between the expression of *NESTIN* and passaging.

In this study, we first observed a significant change in the expression of *NESTIN* in UC-MSCs and the percentage of NESTIN + cells as passaging increased when cells were cultured in the conventional medium (DMEM/F12 with 10% FBS). Flow cytometry analyses for 10th passages revealed that the NESTIN ratio in either FBS or TBD FS groups experienced a significant decline after passage 6. UltraGRO was applied in the UC-MSC culture and the NESTIN^+^ UC-MSCs in the UltraGRO group were maintained at a high percentage (> 90%). The NESTIN mRNA level was also in keeping with this finding. Furthermore, UltraGRO maintained the proliferation capacity of UC-MSCs for up to 10 passages, likely due to certain growth factors present in the UltraGRO supplement (i.e. platelet derived growth factor, transforming growth factor, fibroblast growth factor, insulin like growth factor, etc.), cytokines, and chemokines. UltraGRO is a human single donor platelet derivative collected from healthy donors. Platelet lysate is a better than FBS and serum as a supplement for the ex vivo expansion of MSC (Bernardo et al. 2009; Bieback et al. 2009; Schallmoser et al. 2007). Stem cells proliferate via asymmetric cell division. This proliferation leads to daughter cells inheriting cellular constituents unevenly (Bernal and Arranz 2018; Gomez-Lopez et al. 2014). In addition, major morphological changes, including cytoskeletal modifications that regulate cell polarity, are required for cells to divide asymmetrically. During mitosis, the cytoskeletal protein NESTIN likely regulates the production and destruction of vimentin and other intermediate filaments (Chou et al. 2003). NESTIN may thus contribute to the abilities of self-renewal and proliferation of UC-MSCs, potentially explaining the results of the present study.

The benefit of using UC-MSCs in clinical therapy is their anti-inflammatory and immunomodulatory capabilities in a large range of diseases. UC-MSCs are a good candidate in clinical cellar therapy for treating both acute and chronic inflammatory tissue deterioration in humans and animals. UC-MSCs have immunosuppressive effects in treating Crohn’s disease (CD), one of two major types of inflammatory bowel disease (Feng et al. 2018; Liao et al. 2016). Interestingly, the UC-MSC from the UltraGRO group had better anti-inflammatory performance than in other media. This suggests that the high expression of *NESTIN* in UC-MSC could have better immunomodulatory performance. Further investigation will be required to understand the mechanism of how NESTIN regulates the immunomodulation ability of UC-MSC.

In this study, we monitored the expression of surface markers and NESTIN for up to ten passages under three different culture conditions. Unsurprisingly, the expression of *NESTIN* significantly decreased in late passages using conventional medium (FBS). By comparison, the use of FBS substitute and UltraGRO can retain the expression of *NESTIN*, which barely decreased in the UltraGRO group after ten passages. Furthermore, UC-MSC in UltraGRO group also displays better immunomodulatory performance. However, the role of NESTIN in UC-MSC immunomodulation requires further investigation. It is believed that NESTIN could be a reporter of the status of UC-MSC during the large-scale manufacturing process, which may be used to evaluate the UC-MSC quality in stem cell therapy.

## Data Availability

All relevant data are within the manuscript.

## Abbreviations

MSC: Mesenchymal stem cell
ISCT: International Society of Cell Therapy
cGMP: Current good manufacture practice
UC-MSCs: Umbilical cords-mesenchymal stem cells
DMEM: Dulbecco’s modified Eagle’s medium
FBS: Fetal bovine serum
TNBS: Trinitrobenzene sulfonic acid
CD: Crohn’s disease
